# Knockdown of mediator subunit Med19 suppresses bladder cancer cell proliferation and migration by downregulating Wnt/β‐catenin signalling pathway

**DOI:** 10.1111/jcmm.13229

**Published:** 2017-06-19

**Authors:** Hejia Yuan, Shengqiang Yu, Yuanshan Cui, Changping Men, Diandong Yang, Zhenli Gao, Zhe Zhu, Jitao Wu

**Affiliations:** ^1^ Department of Urology The Affiliated Yantai Yuhuangding Hospital of Qingdao University Yantai Shandong China; ^2^ Department of Stem Cell Biology and Regenerative Medicine Lerner Research Institute Cleveland Clinic Cleveland OH USA

**Keywords:** Med19, proliferation, migration, bladder cancer, Wnt/β‐catenin pathway

## Abstract

Mediator complex subunit 19 (Med19), a RNA polymerase II‐embedded coactivator, is reported to be involved in bladder cancer (BCa) progression, but its functional contribution to this process is poorly understood. Here, we investigate the effects of Med19 on malignant behaviours of BCa, as well as to elucidate the possible mechanisms. Med19 expression in 15 BCa tissues was significantly higher than adjacent paired normal tissues using real‐time PCR and Western blot analysis. Immunohistochemical staining of 167 paraffin‐embedded BCa tissues was performed, and the results showed that high Med19 protein level was positively correlated with clinical stages and histopathological grade. Med19 was knocked down in BCa cells using short‐hairpin RNA. Functional assays showed that knocking‐down of Med19 can suppress cell proliferation and migration in T24, UM‐UC3 cells and 5637 *in vitro*, and inhibited BCa tumour growth *in vivo*. TOP/FOPflash reporter assay revealed that Med19 knockdown decreased the activity of Wnt/β‐catenin pathway, and the target genes of Wnt/β‐catenin pathway were down‐regulated, including Wnt2, β‐catenin, Cyclin‐D1 and MMP‐9. However, protein levels of Gsk3β and E‐cadherin were elevated. Our data suggest that Med19 expression correlates with aggressive characteristics of BCa and Med19 knockdown suppresses the proliferation and migration of BCa cells through down‐regulating the Wnt/β‐catenin pathway, thereby highlighting Med19 as a potential therapeutic target for BCa treatment.

## Introduction

Bladder cancer (BCa) is the second most common cancer arising in the genitourinary tract, and it is characterized by its unpredictable nature of progression 1. Considering that nearly 33–75% of patients failed to respond to therapy due to the disease relapse and metastasis 2, there is still an urgent need for further investigation of the carcinogenesis and development of BCa.

The Mediator is a multiprotein complex that regulates transcription at the level of RNA polymerase II (polII) assembly and links to developmental diseases and cancer 3, 4. Mediator complex subunit 19 (Med19) is one of components of the Mediator complex, which was originally identified in a screening for mutants with increased aerobic expression of the CYC7 gene in yeast 5. An increasing number of studies have identified that Med19 is involved in the progression and metastasis of various cancers 6, 7, 8, 9, 10. Zhang *et al*. 9 first reported the causation of Med19 high expression could facilitate BCa cell growth. In addition, Wen and colleagues suggested that Med19 might promote invasive behaviour and bone metastasis of BCa cells by stimulating the expression of bone morphogenetic protein‐2 (BMP‐2) 11. Although it established the important role of Med19 in BCa, the exact mechanism remains elusive.

Recently, it was reported that the Wnt/β‐catenin signalling pathway is involved in the cell proliferation and differentiation in BCa 12. Mediator complex has been proposed to function as a signal transducer coupled with its genetic links to the Wnt/β‐catenin signalling pathway 13. Therefore, we further performed experiments to test whether Med19 subunit in Mediator plays an important role in BCa cell growth and tumour metastasis *via* regulating Wnt/β‐catenin signalling pathway.

## Materials and methods

### Patient and tissue sample

This study was approved by the Ethics Committee of the Affiliated Yantai Yuhuangding Hospital of Qingdao University (Permit Number: 2013‐46). Each patient provided signed consent to permit the use of samples in our study. We collected 15 fresh BCa tissues paired with corresponding adjacent non‐cancerous tissues from patients who underwent surgery between March 2015 and April 2015. During surgery, fresh tumour tissue and paired non‐cancerous tissue isolated from at least 2 cm away from the tumour border were collected in the operating room and processed immediately in liquid nitrogen within 15 min. None of these patients received neoadjuvant or adjuvant chemotherapy before the operation. In addition, 167 paraffin‐embedded archived BCa samples between July 2013 and February 2015 were obtained from our hospital for immunohistochemistry (IHC). The criteria for enrolment were histopathological identification of bladder urothelial carcinoma, newly diagnosed without preoperative chemotherapy or radiotherapy, and no history of other tumours. All pathology slides were thoroughly re‐evaluated by two senior uropathologists, who were blind to patient clinical outcome. Patients were stratified by gender, and by tumour number, grade, stage and recurrence.

### Immunohistochemical staining and evaluation criteria

All tumour sections were dewaxed and rehydrated by routine methods and incubated in 3% H_2_O_2_ for 30 min. Slides were incubated with rabbit polyclonal primary antibodies against Med19 at a dilution of 1:100 in a humidified chamber 4°C overnight. Sections were stained with 3,3′‐diaminobenzidine (DAB) and counterstained with haematoxylin according to the manufacturer's protocol. Bile duct tissue samples served as negative controls. Sections with confirmed positive expression of Med19 were used as positive controls. Based on the percentage for Med19 immune‐positive tumour cells, a score of one was given when ≤5% of cells were positive; two when 6–25%, three when 26–50% and four when ≥50% of cells were positive. Staining intensity was scored as 0 (negative), 1 (weak), 2 (moderate) and 3 (strong). Both scores were multiplied and the resulting score was used to dichotomize Med19 expression as low (≤6) and high (>6).

### Cell culture and transfection

The human bladder cancer cell lines T24, UM‐UC3 and 5637 were obtained from the Institute of Biochemistry and Cell Biology, Shanghai, China. Cells were grown in RPMI1640 (Gibco BRL, Grand Island, NY, USA) supplemented with 10% foetal bovine serum (Gibco BRL) at 37°C in a humidified incubator with 5% CO_2_. One day prior to infection, cells were plated at a density of 20–30%. Recombinant lentivirus expressing short‐hairpin RNA (shRNA) targeting Med19 (target sequence: shRNA #1, 5′‐GGTGAAGGAGAAGCTAAGT‐3′; shRNA #2, 5′‐GTAGCTCTTTCAATCCTAT‐3′) and a non‐silencing control were constructed by GeneChem, Shanghai, China, and cells were also transfected with the empty vector control. Cells were harvested for analysis of mRNA and protein levels 3 days after infection.

### Cell proliferation assay

Cells were seeded in 96‐well culture plates (3 × 10^3^ cells/well) in triplicates and were examined at 0, 1, 2, 3 and 4 days after incubation. At indicated time‐points, 10 μl (5 mg/ml) of 3‐(4,5‐Dimethylthiazol‐2‐yl)‐2,5‐diphenyltetrazolium bromide (MTT) (Sigma‐Aldrich, St. Louis, USA) was added to each well. After 1‐hr incubation, 150 μl of dimethyl sulfoxide (DMSO) was added for formazan crystals dissolution with a 15‐min incubation time at 37°C. The optical density (OD) was recorded at 490 nm using a microplate reader (Bio‐Rad, Hercules, CA, USA). Cells were seeded into 96‐well plate with 3000 cells/well in triplicate for cell counting at indicated time‐points using Countess II FL Automated Cell Counters (Invitrogen, Carlsbad, CA, USA).

### Wound‐healing assay

Cells (5 × 10^5^) were seeded on six‐well plates and scraped firmly with a plastic pipette tip. The cells were washed once to remove cell debris, and fresh serum‐free medium was added. The wound‐healing process was captured at the beginning, 12 and 24 hrs after scratching. Experiments were carried out in triplicate and repeated three times.

### Transwell migration assay

Polycarbonate membrane inserts with 8‐μm pores (Corning Life Sciences, Bedford, MA, USA) were placed in 24‐well cell culture plates. Cells were suspended at a concentration of 1 × 10^5^ cells/ml in 100 μl of serum‐free medium and were plated in the uncoated upper chamber. Foetal bovine serum (10%), used as a chemoattractant, was added to the bottom chamber. After 24 hrs of incubation, those that had migrated to the bottom surface were fixed, stained and scored visually in five random fields under a microscope. Each experiment was performed in replicate, and the mean value was calculated from three independent experiments.

### Quantitative real‐time reverse transcription PCR (qRT‐PCR)

Total RNA was extracted with TRIzol reagent (Takara, Carlsbad, CA, USA) according to the manufacturer's protocol. The cDNA was synthesized using the Revert Aid First‐Strand cDNA Synthesis Kit (TransGene, Beijing, China). For qRT‐PCR, each sample was analysed in triplicate. The PCR amplification conditions were as follows: 1 cycle at 95°C for 10 min, 45 cycles at 95°C for 15 s and 60°C for 60 s. The relative expression of mRNA was calculated using the formula 2^−∆∆CT^ 12. Table S1 lists the primers.

### Luciferase assay

Wnt signalling activation was measured by transfection with TOPflash/FOPflash reporter plasmids (Addgene, Cambridge, MA, USA). Cells (5 × 10^4^) were seeded in 24‐well plates and cultured for 12 hrs. Cells were transfected with 100 ng of TOPflash/FOPflash reporter luciferase plasmid and 5 ng of the internal control plasmid pRL‐TK (Progema, Madison, WI, USA) using Lipofectamine 2000 (Invitrogen). The luciferase activity was measured 36 hrs after transfection by Luciferase Assay System (Promega) according to the manufacturer's protocol, and the firefly luciferase activity was normalized to the renilla luciferase activity. All experiments were performed three times in triplicate.

### Western blot

Protein lysates were loaded to each lane for analysis by 12% sodium dodecyl sulphate–polyacrylamide gel electrophoresis (SDS‐PAGE) and were subsequently transferred to nitrocellulose (NC) membranes (Millipore, Darmstadt, Germany). After blocking with 5% non‐fat milk in Tris‐buffered saline with Tween for 1 hr, the membrane was incubated with primary antibody at 4°C overnight. Additional information about the primary antibodies and their dilutions is provided in Table 1. After three times of 10‐min washes with Tris‐buffered saline with Tween, the membrane was incubated with HRP‐conjugated secondary antibodies (1:5000) (Santa Cruz, CA, USA) for 1 hr at room temperature. Immunoreactive bands were detected by a chemiluminescent detection system (ECL, Pierce, Rockford, IL, USA). The relative protein levels were calculated based on β‐actin protein.

**Table 1:** Antibody and dilution

Antibody	Biological source	Clone	Dilution for Western blot	Company
Anti‐Med19	Rabbit	Polyclonal	1:200	Sigma‐Aldrich, St. Louis, USA
Anti‐Wnt2	Rabbit	Polyclonal	1:200	Santa Cruz, CA, USA
Anti‐β‐catenin	Mouse	Monoclonal	1:1000	Millipore, Darmstadt, Germany
Anti‐E‐cadherin	Mouse	Monoclonal	1:200	Abcam, Cambridge, UK
Anti‐Gsk3β	Rabbit	Monoclonal	1:5000	Abcam, Cambridge, UK
Anti‐Cyclin‐D1	Rabbit	Polyclonal	1:200	Santa Cruz, CA, USA
Anti‐MMP‐9	Rabbit	Polyclonal	1:500	Santa Cruz, CA, USA
Anti‐β‐actin	Mouse	Monoclonal	1:2000	Santa Cruz, CA, USA

### Xenograft model of tumour growth *in vivo*

The experimental procedures were approved by the hospital institutional animal care and use committee. Male immune‐deficient BALB/c nude mice (4–6 weeks old) were purchased from Beijing Wei‐tong Li‐hua Laboratory Animals and Technology Ltd, Beijing, China. The cells (1 × 10^6^) were suspended in Matrigel (BD Biosciences, Franklin Lakes, NJ, USA) and subcutaneously implanted into the left flank (control cells) and right flank (shMed19‐transfected cells) of nude mice. Fifteen days after implantation, tumour volumes were measured every 5 days until the mice were killed at day 30. Tumour mass was weighted and tumour volume was calculated using the following formula: *V* (mm^3^) = 1/2 length^2^ × width^2^. Proliferative index was investigated using IHC staining for Ki‐67, and the experimental procedure was performed as above.

### Statistical analysis

All statistical analyses were performed by SPSS 19.0 (Chicago, IL, USA). Data are presented as mean ± SD of three independent experiments. Differences between groups were analysed with Student's *t*‐test for unpaired observations. Chi‐square test was used to evaluate the results of IHC staining. Mann–Whitney *U*‐test was used to compare the Western blot results of Med19 expression in 15 paired BCa and the adjacent normal tissues. Statistical significance was considered if *P* < 0.05.

## Results

### Correlation between Med19 expression and clinicopathological parameters

**Figure 1:**
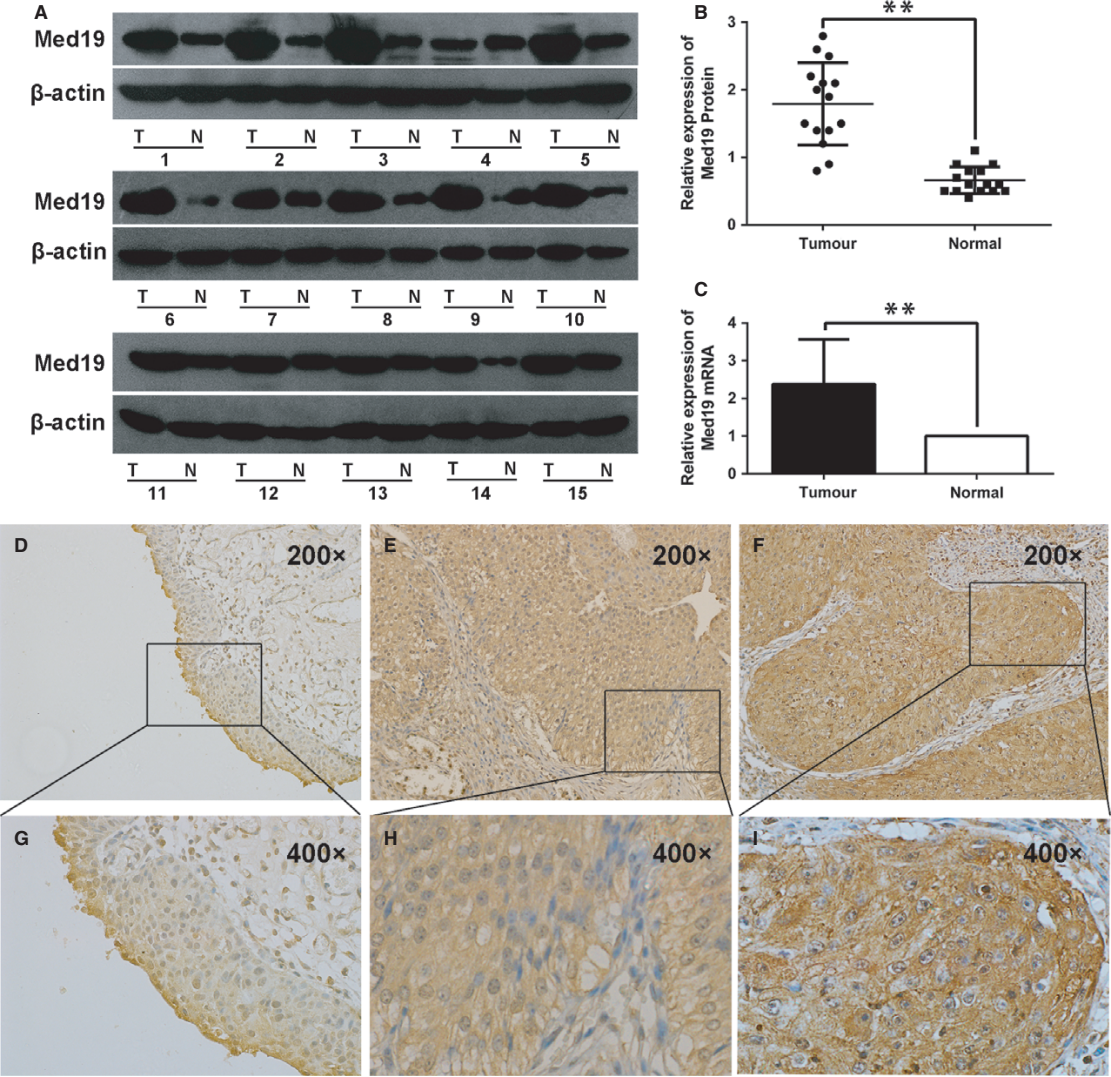
The expression of Med19 in human BCa. (**A** and **B**) Western blot showed Med19 protein level in 15 pairs of human BCa tumour tissues (T) and adjacent normal tissues (N). (**C**) qRT‐PCR assay showed similar expression pattern of Med19 mRNA level. (**D**–**I**) Detection of Med19 protein by IHC. (**D** and **G**) Normal bladder tissue. (**E** and **H**) Low‐grade BCa tissue. *F* and *I*, high‐grade BCa tissue. *P < 0.05, **P < 0.01.

We examined Med19 protein expression in 15 paired BCa tissues and the adjacent normal tissues by Western blot analysis. Med19 has higher expression level in BCa tissues than in the adjacent normal tissues (*P* < 0.01, Fig. 1A and B), and similar result was observed on the mRNA level of Med19 by qRT‐PCR (*P* < 0.01, Fig. 1C). In normal tissues, Med19 protein staining was very weak (Fig. 1D and G). However, the staining of Med19 was moderate in low‐grade tumour tissues (Fig. 1E and H) and strong in high‐grade ones (Fig. 1F and I). Table 2 summarizes the association between Med19 protein expression and clinicopathological characteristics. Med19 overexpression was detected in 88 of 167 cases (52.7%). The expression level of Med19, particularly localized in the nucleus and cytoplasm of tumour cells, correlates with advanced stage (*P* = 0.003) and high grade (*P* = 0.001), but did not show a significant association with gender (*P* = 0.738), tumour number (*P* = 0.071) or recurrence (*P* = 0.101).

**Table 2:** Association of Med19 expression with clinical pathologic characteristics of patients

Parameters	Number	Med19 expression		*P* value
		Low (%)	High (%)	
Patients	167	79 (47.3)	88 (52.7)	
Gender				0.738
Male	127	61 (48.0)	66 (52.0)	
Female	40	18 (45.0)	22 (55.0)	
Stage				0.003
≤T1	96	55 (57.3)	41 (42.7)	
>T1	71	24 (33.8)	47 (66.2)	
Grade				0.001
1	42	30 (71.4)	12 (28.6)	
2	65	28 (43.1)	37 (56.9)	
3	60	21 (35.0)	39 (65.0)	
Tumour number				0.071
Unifocal	120	62 (51.7)	58 (48.3)	
Multifocal	47	17 (36.2)	30 (63.8)	
Recurrence				0.101
Positive	116	50 (43.1)	66 (56.9)	
Negative	51	29 (56.9)	22 (43.1)	

### Knockdown of Med19 inhibited bladder cancer cell growth *in vitro* and *in vivo*

**Figure 2:**
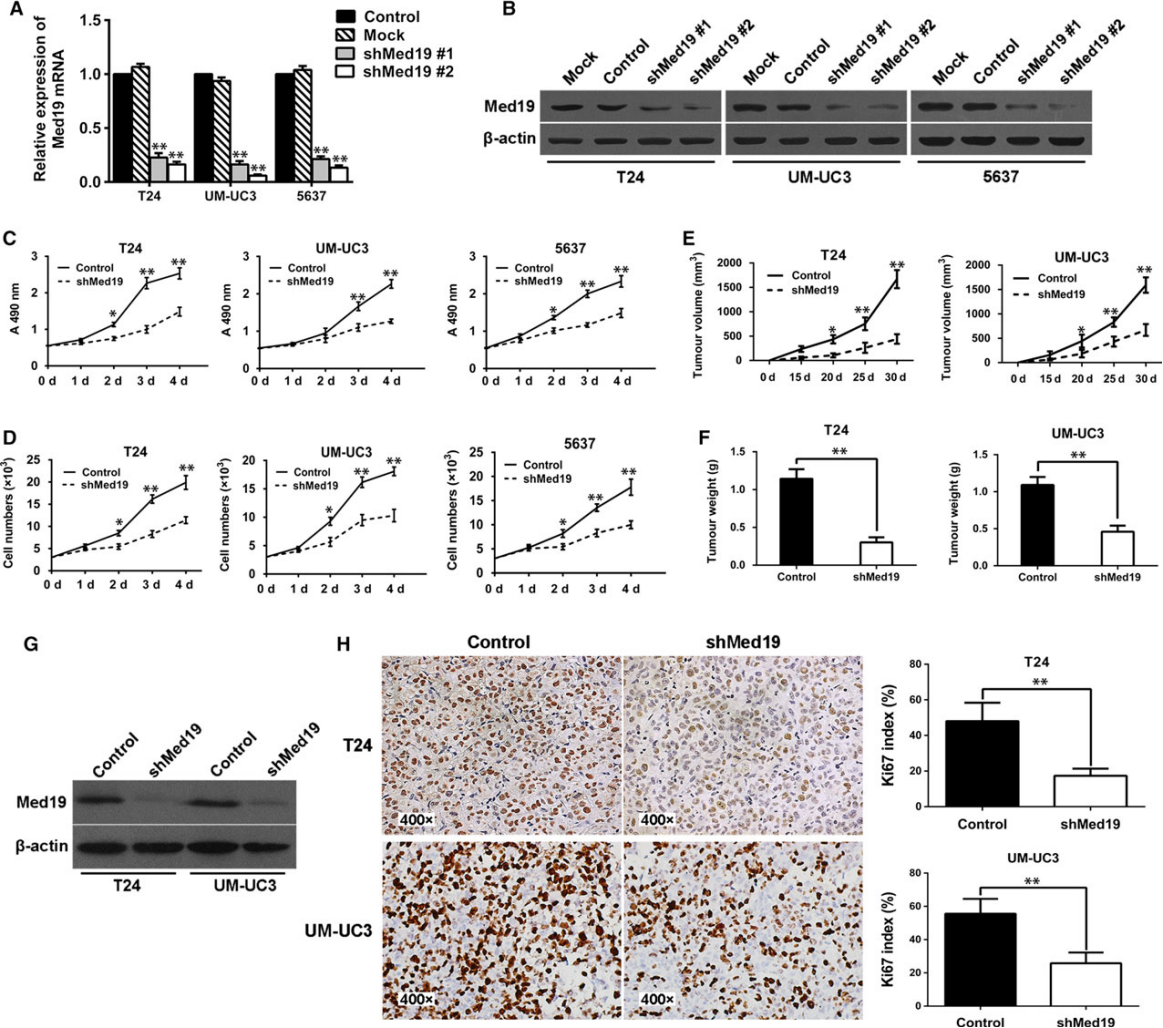
Knockdown of Med19 inhibited BCa cell growth *in vitro* and *in vivo*. Cells were transfected with shRNA targeting Med19 (shMed19), non‐silencing control shRNA (Control) and empty vector (Mock). (**A** and **B**) Med19 knockdown efficiency was detected by qRT‐PCR and Western blot. (**C** and **D**) Med19 knockdown decreased cell proliferation ability in T24, UM‐UC3 and 5637 cells. (**E**) Average volume of implanted tumour in each group shown by serial curve measurements. (**F**) Med19 knockdown decreased the tumour weight. (**G**) detection of Med19 protein in tumour xenografts by Western blot. (**H**) IHC analysis of Ki‐67 expression in tumour samples. *P < 0.05, **P < 0.01.

To examine the functional role of Med19 in BCa, shRNA‐mediated knockdown of Med19 was achieved by lentiviral transduction of T24, UM‐UC3 and 5637 cells, which was demonstrated by green fluorescent protein (GFP) expression under fluorescence microscopy. The qRT‐PCR results confirmed two specific sequences against Med19 (shMed19, #1 and #2) significantly inhibited Med19 mRNA expression in T24, UM‐UC3 and 5637 cells (Fig. 2A). We further confirmed the knockdown effect of Med19 at protein level by Western blot (Fig. 2B). These results suggested successful knockdown of endogenous Med19 expression by Med19 shRNAs in T24, UM‐UC3 and 5637 cells.

To evaluate the viability of BCa cells *in vitro*, MTT assay was employed and a strong inhibition of cell proliferation, reflected by the optical density, was observed in the Med19 shRNA‐treated groups compared to the control groups (*P* < 0.05, Fig. 2C). Comparing the growth curves of BCa cells, we also found that Med19 knockdown by shRNA significantly reduced cell proliferation rates over 4 days *in vitro* (*P* < 0.05, Fig. 2D). To test the functional role of Med19 in tumour growth *in vivo*, T24 and UM‐UC3 cells expressing shRNA targeting Med19 or control shRNA were subcutaneously inoculated into nude mice. Thirty days later, tumours were harvested, and measurement of tumour volume showed that knocking‐down of Med19 protein leads to lower tumour volume (both *P* < 0.05, Fig. 2E) and reduced tumour weight compared to control groups (both *P* < 0.01, Fig. 2F). Furthermore, IHC analysis showed that down‐regulation of Med19 suppressed the expression of proliferation marker Ki‐67 in tumour tissues compared with control shRNA‐treated groups (Fig. 2H). Results of quantitative analysis of Ki‐67‐positive cells (index) in the tumour are summarized in Figure 2H. As compared with the controls, the Ki‐67 index in the tumour tissues was significantly decreased after the Med19 shRNA treatment (both *P* < 0.01). These findings were consistent with the results of the cell proliferation assay *in vitro*.

### Knockdown of Med19 inhibited bladder cancer cell migration *in vitro*

**Figure 3:**
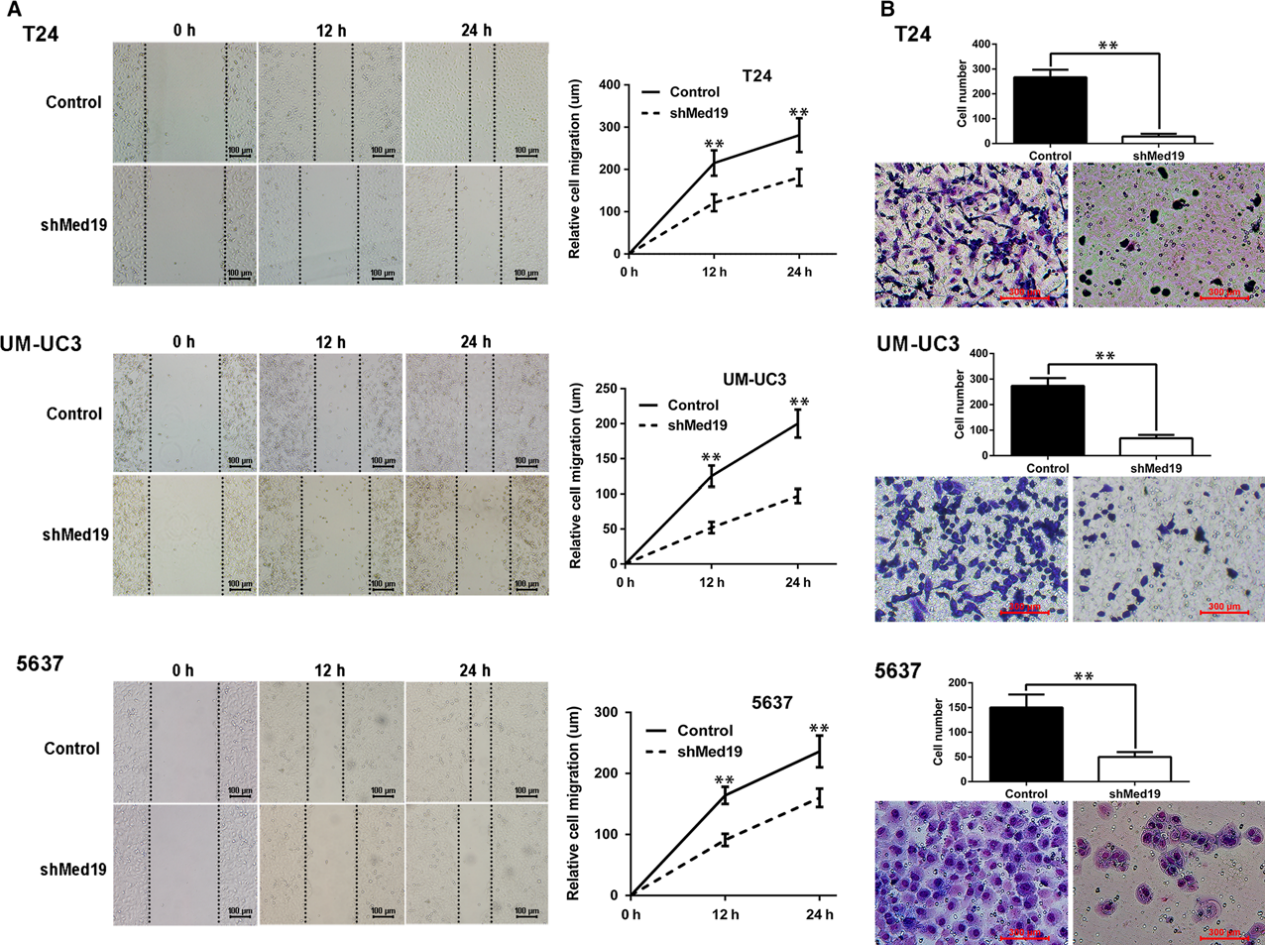
Knockdown of Med19 inhibited BCa cell migration *in vitro*. (**A**) wound‐healing assay showed that shMed19‐treated cells migrated significantly slower than the control groups. (**B**) transwell migration assay, the upper panel showed the migrated cells and the lower panel showed cell numbers. The average migrated cell numbers of shMed19 treatment were significantly decreased compared to controls. *P < 0.05, **P < 0.01.

Cell migration ability is a critical parameter for metastatic potential of cancer cells. To test whether Med19 contributes to BCa cell migration, wound‐healing and transwell migration assays were performed for cells expressing Med19 shRNA or control shRNA. For wound‐healing assay, 24 hrs after the scratch, the gaps in the control groups were apparently smaller than the ones in the Med19 shRNA groups (Fig. 3A), indicating the role of Med19 in BCa cell migration. Suppression of cell migration in Med19 shRNA‐expressing cells was also observed by the transwell assay, where knockdown of Med19 reduced the number of T24, UM‐UC3 and 5637 cells that migrated into the lower filter (all *P* < 0.01, Fig. 3B).

### Med19 knockdown stimulated E‐cadherin expression and suppressed activity of Wnt/β‐catenin pathway

**Figure 4:**
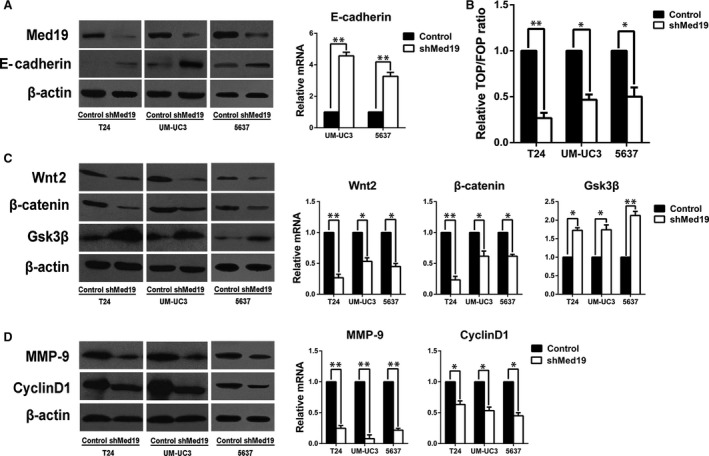
Med19 knockdown stimulated E‐cadherin expression, suppressed Wnt/β‐catenin pathway. (**A**) E‐cadherin expression levels were significantly up‐regulated in shMed19 groups. (**B**) Activities of Wnt/β‐catenin signalling were decreased in shMed19 groups by TOP/FOP luciferase reporter assay. (**C**) Wnt2 and β‐catenin expression levels were significantly down‐regulated, and Gsk3β was up‐regulated in shMed19 groups. (**D**) both MMP‐9 and Cyclin‐D1 expression levels were significantly down‐regulated in shMed19 groups. β‐actin was used as internal control. *P < 0.05, **P < 0.01.

Given the effects of Med19 on proliferation and migration of BCa cells, we looked into E‐cadherin and Wnt/β‐catenin pathway, which plays key roles in cell proliferation and migration, and we analysed the expression of E‐cadherin and the activity of Wnt/β‐catenin pathway. We observed an increased protein level of E‐cadherin after Med19 knockdown (Fig. 4A). TOP/FOPflash luciferase reporter assay was used to examine the effect of Med19 on β‐catenin/TCF‐dependent transcriptional activity, as a canonical experiment for the detection of Wnt/β‐catenin signalling activity 14. The TOP/FOPflash reporter activities in T24, UM‐UC3 and 5637 cells with Med19 knockdown by shRNA were significantly inhibited (all *P* < 0.05, Fig. 4B). In addition, depletion of Med19 reduced the protein expression of Wnt2 and β‐catenin. However, protein level of Gsk3β was elevated (*P* < 0.05, Fig. 4C). We then examined the protein expression levels of matrix metalloproteinase‐9 (MMP‐9) and Cyclin‐D1, which are classic downstream targets of the Wnt/β‐catenin signalling pathway 15, 16, 17, 18. Indeed, depletion of Med19 had a potent inhibitory effect on the expression of MMP‐9 and Cyclin‐D1 (*P* < 0.05, Fig. 4D). We also confirmed these data at mRNA level (Fig. 4A, C and D). Therefore, these results suggest that Med19 knockdown stimulated E‐cadherin expression and decreased the activity of Wnt/β‐catenin signalling pathway, possibly leading to the suppression of BCa cell proliferation and migration.

### Inhibition of BCa cell proliferation and migration by Med19 knockdown can be rescued by activating Wnt/β‐catenin signalling

**Figure 5:**
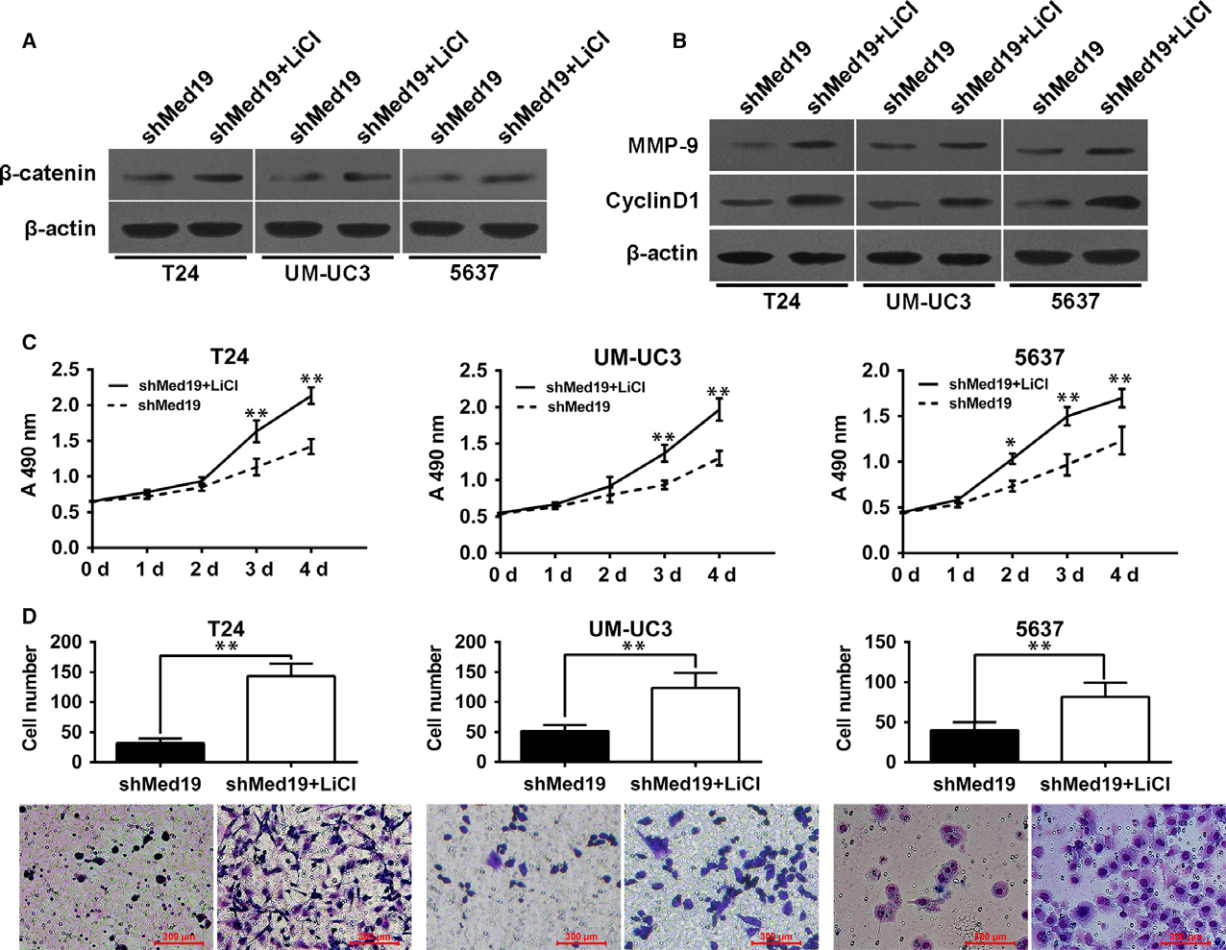
Inhibition of BCa cell proliferation and migration by Med19 knockdown can be rescued by LiCl. (**A**) BCa cells were treated with or without 10 mM LiCl, the protein expression of β‐catenin was examined by Western blot. (**B**) Both MMP‐9 and Cyclin‐D1 protein levels were increased in shMed19 cells treated with LiCl. (**C**) Cell proliferation assay evaluated the effect of LiCl in BCa cells of shMed19 treatment. (**D**) transwell migration assay evaluated the effect of LiCl in BCa cells of shMed19 treatment. β‐actin was used as internal control. *P < 0.05, **P < 0.01.

To further confirm the role of Med19 in regulating the activity of the Wnt/β‐catenin signalling, lithium chloride (LiCl, sigma) was used to activate the Wnt/β‐catenin pathway. As demonstrated in Fig. 5A, LiCl significantly increased protein level of β‐catenin in Med19 shRNA‐treated cells. As expected, LiCl markedly increased the expression of Cyclin‐D1 and MMP‐9 in BCa cells along with Med19 knockdown (Fig. 5B). Furthermore, we examined the biological mechanism of Med19 in BCa cells. Through MTT and transwell assay, we found that activation of Wnt/β‐catenin signalling by LiCl promoted cell proliferation and migration in Med19 shRNA‐treated BCa cells (Fig. 5C and D). Collectively, our results indicate that Wnt/β‐catenin signalling is a functional mediator of Med19‐related proliferation and migration in BCa cells.

## Discussion

Med19 is a key subunit of the mammalian Mediator complex. Due to its critical role in stabilizing the entire Mediator complex, Med19 is indispensable for the regulation of gene transcription. In the absence of Med19, the binding affinity of the Mediator with RNA polymerase II is decreased and phosphorylation of the carboxyl terminal domain (CTD) *via* the transcription factor II H fails to occur 19, 20. Med19 also plays an important role in tumour formation and progression. Wen and coworkers suggested that Med19 might promote invasive behaviour and bone metastasis of BCa cells by stimulating the expression of BMP‐2 11. In breast cancer, osteosarcoma and hepatocellular carcinoma, cell proliferation capability was significantly reduced *in vitro* and *in vivo* following knockdown of Med19 6, 8, 10. These results indicate that Med19 is a human oncogene that might represent a promising new target for therapeutic intervention.

The present study demonstrates that Med19 is highly expressed in BCa tissues and Med19 expression levels are positively correlated with clinical stage and pathological grade of BCa (Table 2). It is concluded that increased expression of Med19 might promote BCa development. We then applied a lentivirus‐mediated shRNA targeting assay to knockdown Med19 in BCa cells. The results showed that BCa cell proliferation capacity was remarkably decreased *in vitro* and *in vivo* following Med19 knockdown, which might be due to the down‐regulation of Cyclin‐D1 signalling, an important regulator for cell cycle progression 21. In addition, Med19 inhibition can significantly suppress BCa cell migration by wound‐healing and transwell migration assays (Fig. 3A and B). Interestingly, the down‐regulation of MMP‐9 and up‐regulation of E‐cadherin in our results may explain why depletion of Med19 significantly impaired BCa cell migration capacity. In line with previous findings, both increased expression of MMP‐9 and decreased expression of E‐cadherin correlate with bladder cancer metastasis 22, 23.

The Wnt signalling pathway regulates cell proliferation and differentiation, and activation of Wnt pathway is involved in the pathogenesis of BCa 23. Mediator complex is commonly seen as a molecular bridge that connects DNA‐binding transcription factors to RNA polymerase II, which regulate different signalling events into the appropriate response at level of RNA transcription 3, 4, 24. Some subunits of Mediator complex have been shown to play an essential role in several important signalling pathways related to carcinogenesis, including TGF‐β/SMAD, AKT/MAPK and NF‐κB signalling 25, 26. Subunit Med12 interacts with β‐catenin, and it has been identified as an important transducer of Wnt/β‐catenin signalling in the development of colorectal tumours 27. Imberg‐Kazdan *et al*. 7 recently identified Med19 as a regulator that affects androgen receptor (AR) signals and proliferation of AR‐expressing prostate cancer. However, the mechanism Med19 interacts with other signalling pathways leads to cancer development remains poorly understood, and further investigation is needed to determine the function of Med19 in BCa initiation and progression.

Aberrant activation of the Wnt/β‐catenin signalling pathway, resulting in uncontrolled cell proliferation and impaired migration, is a common event in human cancer development 23, 28. Wnt/β‐catenin signalling activation inhibits glycogen synthase kinase‐3β (GSK3β) phosphorylation of β‐catenin, leading to its cytosolic accumulation 28. It has been shown that E‐cadherin inhibits the Wnt/β‐catenin pathway through its binding at C‐terminal domain of β‐catenin on the cellar surface, thereby sequestering β‐catenin from the cytoplasmic pool 29. We found the suppression of Med19 enhanced expression of E‐cadherin by both qRT‐PCR and Western blot assays. In the Wnt pathway, β‐catenin acts as a key transcriptional coactivator and transmits extracellular signals for the activation of important downstream target genes such as MMP‐9 and Cyclin‐D1 15, 16, 17, 18, 30, 31. Our results showed that the expression of Wnt2 and active β‐catenin, core members of the Wnt/β‐catenin signalling pathway, were decreased following Med19 knockdown, and the expression of GSK3β, a molecule indispensable for maintaining low levels of β‐catenin, was significantly increased. Furthermore, MMP‐9 and Cyclin‐D1 significantly decreased after attenuation of Med19. To further confirm the role of Med19 in regulating the activity of the Wnt/β‐catenin pathway, LiCl was used to activate the Wnt/β‐catenin pathway. Our data showed that the inhibition of BCa cell proliferation and migration by Med19 knockdown can be rescued by cells treated with LiCl. TOP/FOPflash luciferase reporter assay also confirmed that Med19 knockdown inhibited the activation of Wnt/β‐catenin pathway. Together, these data indicate that Med19 participates in BCa cell survival through modulating the Wnt/β‐catenin signalling pathway.

Mediator complex can be divided into four different modules termed the head, middle, tail and cyclin‐dependent kinase 8 (CDK8) module 3, 4. Med19 is a component of head module and plays an important role in regulating gene expression as a transcription coactivator. Our finding shows that Med19 expression affects the BCa cell proliferation and migration, which correlates with the Wnt/β‐catenin pathway. To the best of our knowledge, this is the first report to present effects of Med19 on cancer progression induced by Wnt/β‐catenin pathway. However, the mechanism of how Med19 interacts with Wnt/β‐catenin pathway is still unclear; thus, it will be necessary to perform further investigations in future studies.

In conclusion, our study suggests that high expression of Med19 is closely associated with aggressive characteristics of BCa, and provide good evidence to show the involvement of Med19 in both proliferation and migration of BCa cells, which directly regulates Wnt/β‐catenin signalling pathway. These findings strongly indicate that Med19 may act as an oncogene in BCa development, thereby highlighting Med19 as a potential therapeutic target for BCa treatment.

## Conflict of interest

The authors confirm that there are no conflict of interests.

## Author contribution

Hejia Yuan and Jitao Wu designed and conducted the experiments and analysed data. Shengqiang Yu was involved in the design, animal experiment and drafting of the manuscript. Yuanshan Cui and Changping Men collected the data of patients with bladder cancer. Diandong Yang performed Western blot and participated in the animal experiment. Hejia Yuan and Zhenli Gao were responsible for cell culture. Zhe Zhu and Jitao Wu wrote and revised the manuscript.

## Supporting information


**Table S1** Primer sequence for qRT‐PCR
