# Corrigendum

**DOI:** 10.1111/jcmm.17207

**Published:** 2022-05-07

In Hejia Yuan et al.,^1^ the wound healing picture of the bladder cancer cells 5637 from the group of shMed19 in Figure 3A is incorrect. The correct figure is shown below. The authors confirm all results and conclusions of this article remain unchanged.

**FIGURE 3:**
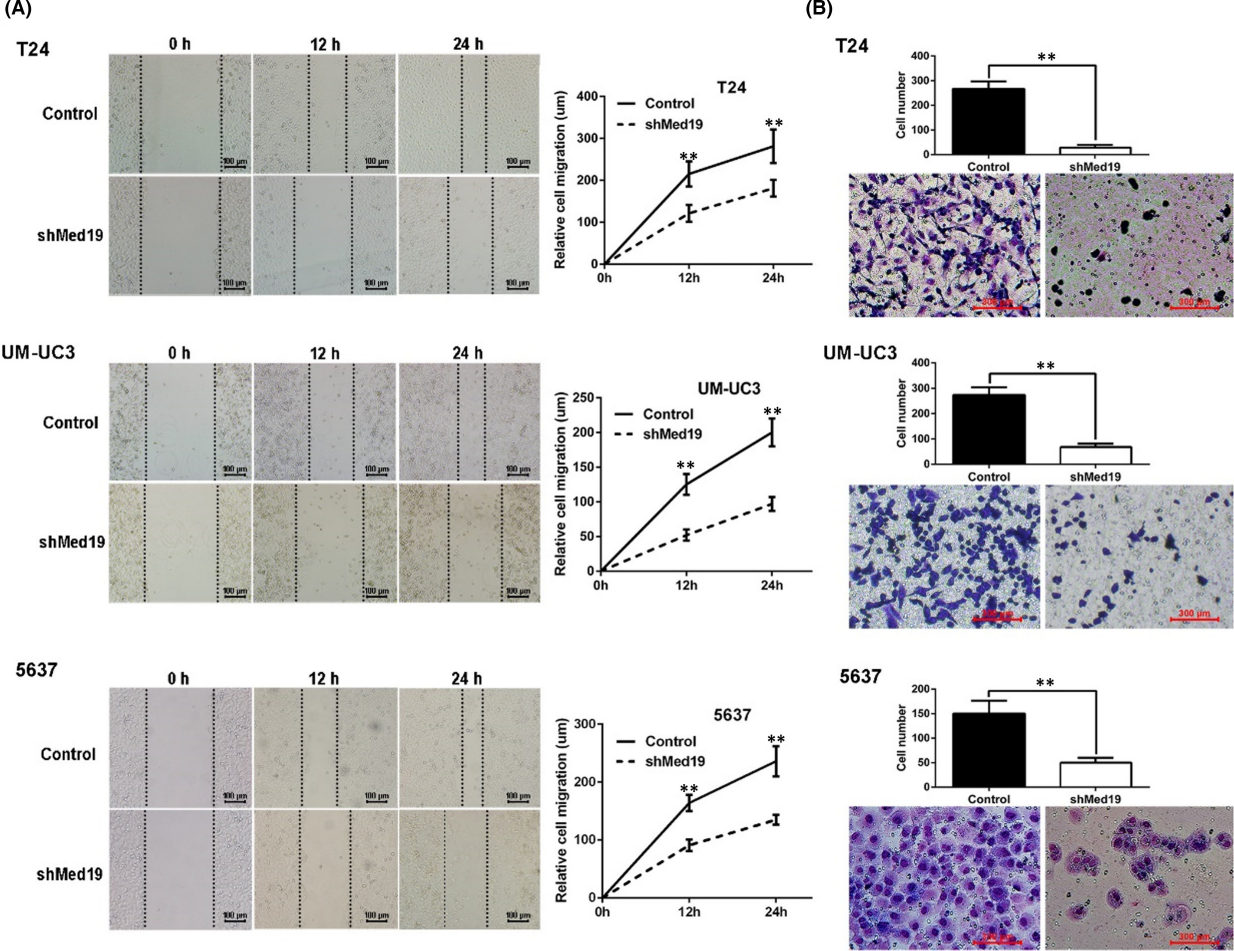
Knockdown of Med19 inhibited BCa cell migration in vitro. (A) wound‐healing assay showed that shMed19‐treated cells migrated significantly slower than the control groups. (B) transwell migration assay, the upper panel showed the migrated cells and the lower panel showed cell numbers. The average migrated cell numbers of shMed19 treatment were significantly decreased compared with controls. *p < 0.05, **p < 0.01

Also, the Western band of the loading control in Figure 5A is incorrect. The correct figure is shown below. The authors confirm all results and conclusions of this article remain unchanged.

**FIGURE 5:**
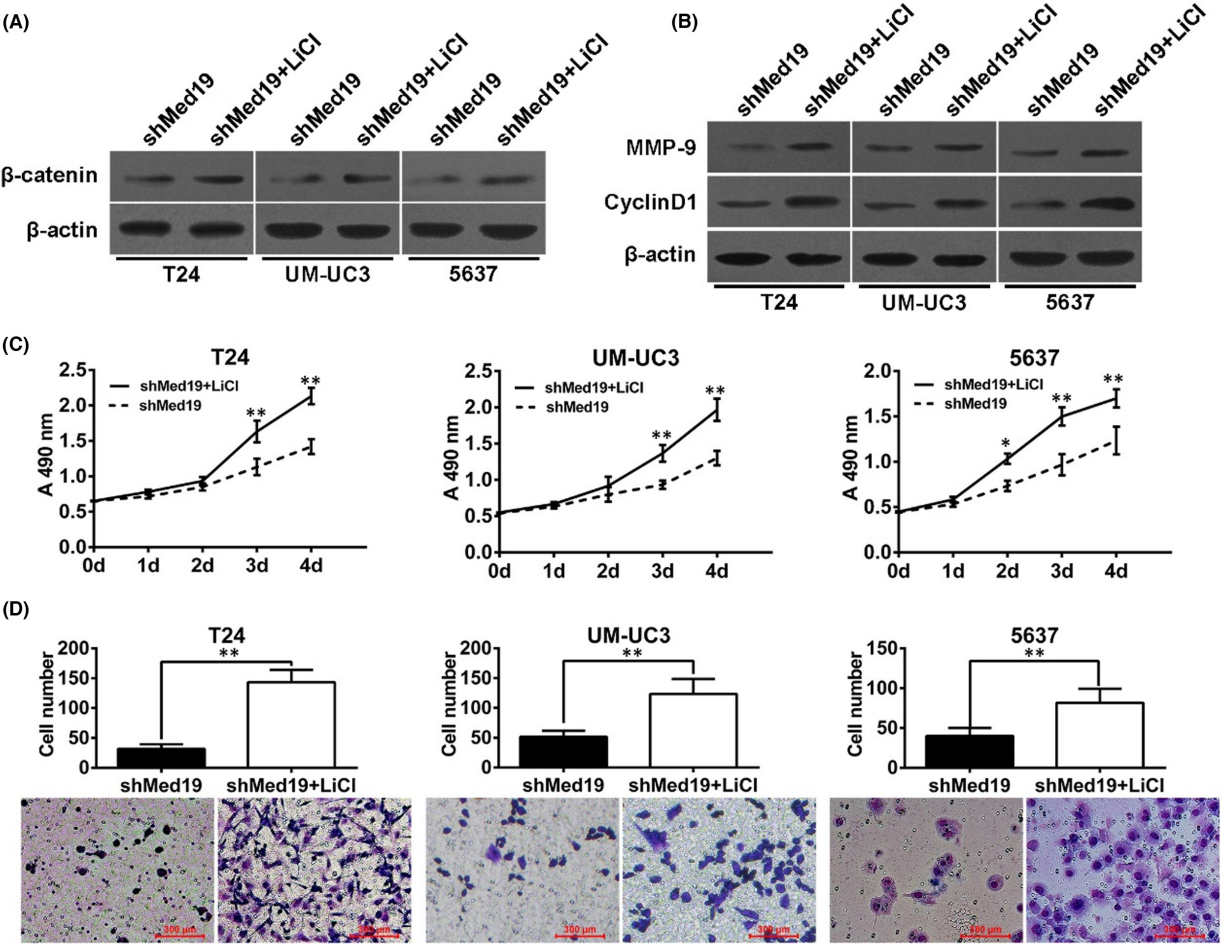
Inhibition of BCa cell proliferation and migration by Med19 knockdown can be rescued by LiCl. (A) BCa cells were treated with or without 10 mM LiCl, the protein expression of b‐catenin was examined by Western blot. (B) Both MMP‐9 and Cyclin‐D1 protein levels were increased in shMed19 cells treated with LiCl. (C) Cell proliferation assay evaluated the effect of LiCl in BCa cells of shMed19 treatment. (D) Transwell migration assay evaluated the effect of LiCl in BCa cells of shMed19 treatment. b‐actin was used as internal control. *p < 0.05, **p < 0.01
