# Supplementary material for: Knockdown of mediator subunit Med19 suppresses bladder cancer cell proliferation and migration by downregulating Wnt/β‐catenin signalling pathway
Source: J Cell Mol Med. 2017 Jun 19;21(12):3254–63. doi: 10.1111/jcmm.13229 (PMC5706513; doi:10.1111/jcmm.13229)
Supplement: Supplementary file 1 — Table S1 Primer sequence for qRT‐PCR [file JCMM-21-3254-s001.docx]

| **Primer** | **Forward (5' - 3')** | **Reverse (5' - 3')** |
| --- | --- | --- |
| Med19 | TGCCAGGGATGATTGATCTG | TCTTCTTGGGAGGCTGAATATG |
| Wnt2 | CGGGAATCTGCCTTTGTTTATG | TTGGATCACAGGAACAGGATTT |
| β-catenin | GGACCAGGTGGTGGTTAATAAG | CATCTGAGGAGAACGCATGATAG |
| E-cadherin | CTCCCAATACATCTCCCTTCAC | AGGTGGTCACTTGGTCTTTATT |
| Gsk3β | TGGAGCCACTGATTATACCTCTA | CCAACTGATCCACACCACTATC |
| CyclinD1 | GCGGAGGAGAACAAACAGAT | GAGGGCGGATTGGAAATGA |
| MMP-9 | GAACTTTGACAGCGACAAGAAG | CGGCACTGAGGAATGATCTAA |
| β-actin | GGCGGCACCACCATGTACCCT | AGGGGCCGGACTCGTCATACT |

**Supplementary Table 1.** Primer sequence for qRT-PCR
